# Supplementary material for: CRISPR/Cas9-Mediated Knockout of OsbZIP76 Reveals Its Role in ABA-Associated Immune Signaling in Rice
Source: Int J Mol Sci. 2025 Jul 2;26(13):6374. doi: 10.3390/ijms26136374 (PMC12249981; doi:10.3390/ijms26136374)
Supplement: Supplementary file 1 [file ijms-26-06374-s001.zip › ijms-3699150-supplementary.pdf]

Supplementary Data

CRISPR/Cas9-Mediated Knockout of *OsbZIP76* Reveals Its Role in ABA-Associated Immune Signaling in Rice

Yu-Jin Jung [1,2], Jin-Young Kim [1], Yong-Gu Cho [3] and Kwon Kyoo Kang [1,2,*]

Contents

Supplementary Figure S1. Generation and molecular confirmation of *OsbZIP76* knockout rice lines via *Agrobacterium*-mediated transformation. (A) Plant regeneration from transformed calli. Embryogenic rice calli were co-cultivated with *Agrobacterium tumefaciens* harboring a CRISPR/Cas9 vector targeting *OsbZIP76*. Transformed calli were selected on medium containing phosphinothricin (PPT) and subsequently regenerated. The red-circled callus represents a successfully selected line that regenerated into a mature transgenic plant. (B) PCR confirmation of T-DNA insertion using nos/bar-specific primers. Genomic DNA from PPT-resistant putative transformants was used to amplify the nos/bar cassette, confirming the presence of the transgene in multiple independent lines.

Supplementary Table S1. sgRNA designed in this study.

Supplementary Table S2. The primers list used in this study.

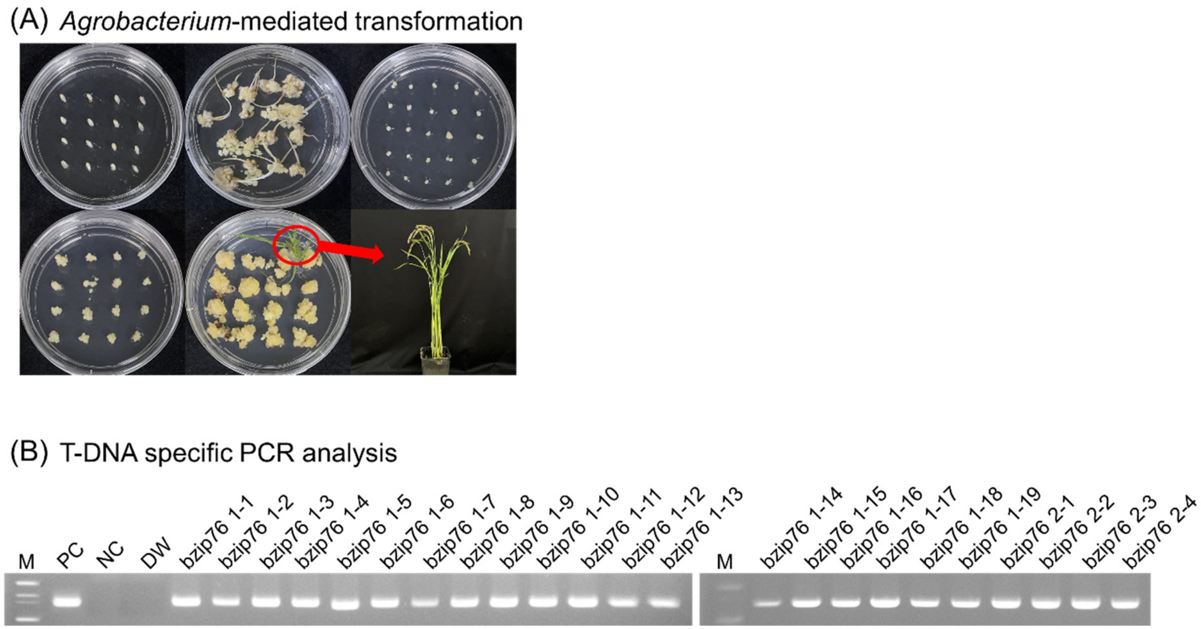

Supplementary Figure S1. Generation and molecular confirmation of *OsbZIP76* knockout rice lines via *Agrobacterium*-mediated transformation. (A) Plant regeneration from transformed calli. Embryogenic rice calli were co-cultivated with *Agrobacterium tumefaciens* harboring a CRISPR/Cas9 vector targeting *OsbZIP76*. Transformed calli were selected on medium containing phosphinothricin (PPT) and subsequently regenerated. The red-circled callus represents a successfully selected line that regenerated into a mature transgenic plant. (B) PCR confirmation of T-DNA insertion using nos/bar-specific primers. Genomic DNA from PPT-resistant putative transformants was used to amplify the nos/bar cassette, confirming the presence of the transgene in multiple independent lines.

Supplementary Table S1. sgRNA designed in this study.

Gene Name	RGEN Target (5' to 3')	Direction	GC Contents (%, w/o PAM)	Out-of-frame Score	Mismatches			
					0	1	2	3
OsbZIP76 sg1	CGTTCTCGGTGGTCTCGATACGG	-	55	83.5	1	0	0	0
OsbZIP76 sg2	ATAGTTCAACCTGCGCTCCTTGG	-	50	61.8	1	0	0	0

Supplementary Table S2. The primers list used in this study.

Primer name	Sequence (primer direction 5′-3′)	purpose
J67 pBOsC sgSEQ - FW	CAGCTTGGCTCTAGTCGACC	Vector construction
K20 RGEN scaaffold regoin RV	CGGTGCCACTTTTTCAAGTT	
OsbZIP76 sg1 up	ggcagCGTTCTCGGTGGTCTCGATACGG	
OsbZIP76 sg1 down	aaacCCGTATCGAGACCACCGAGAACGc	
OsbZIP76 sg2 up	ggcagATAGTTCAACCTGCGCTCCTTGG	
OsbZIP76 sg2 down	aaacCCAAGGAGCGCAGGTTGAACTATc	
T-DNA confirm-Nos ter Fw	TTGCGCGCTATATTTTGTTTT	T-DNA confirm
T-DNA confirm-Bar R Rv	CGTCAACCACTACATCGAGA	
OsbZIP76 sg1 1st F1	GTAGGGTTTCCACAGCCAAA	
*OsbZIP76 sg1*1st R1	ACTCGCCATGATCGTCTTCT	
OsbZIP76 sg1 2nd F1	ACACTCTTTCCCTACACGACGCTCTTCCGATCTTCAAGTTTGAAAGCTGCATT	
OsbZIP76 sg1 2nd R1	GTGACTGGAGTTCAGACGTGTGCTCTTCCGATCTACAACGGGTCCATTTCCA	
OsbZIP76 sg2 1st F1	CGAAGAAGGTCAAGAGGTGA	
OsbZIP76 sg2 1st R1	GTTCCCTACCCGTTTCTTGT	
OsbZIP76 sg2 2nd F1	ACACTCTTTCCCTACACGACGCTCTTCCGATCTGGACCTGATGGTGTCTGTTG	
OsbZIP76 sg2 2nd R1	GTGACTGGAGTTCAGACGTGTGCTCTTCCGATCTTCCAAATCAGAATGACCACA	
OsACTIN FW	CAACACCCCTGCTATGTACG	qRT-PCR analysis
OsACTIN RV	ATCACCAGAGTCCAACACAA	

OsbZIP76 qRT-PCR Fw	AAGGAGCGCAGGTTGAACTA
OsbZIP76 qRT-PCR Rv	TTTCATCTGGCGTTCCCTAC
OsPR1a(Os01g28450) qRT-PCR Fw	TGAGAAGCACTCATGGATCG
OsPR1a(Os01g28450) qRT-PCR Rv	TGATCATCAGCCTCAAATGC
OsPR5(Os12g36880) qRT-PCR Fw	AAGTTGCACTGTGAGCTTAGGA
OsPR5(Os12g36880) qRT-PCR Rv	TTCACGTTTTACTGCGACAGA
OsNPR1(Os01g09800) qRT-PCR Fw	GCTCTGTGCCACCTACCATT
OsNPR1(Os01g09800) qRT-PCR Rv	ACAGCCACAAGCAACATGAG
